# Duloxetine ameliorates acute pancreatitis-associated inflammation and modulates pain related neuroinflammatory pathways via modulation of SP-related neurokinin signaling and PAR2/TRPV1 pathways

**DOI:** 10.3389/ftox.2026.1891784

**Published:** 2026-08-07

**Authors:** Khaled H. Almutary, Ahmed R. Elsheakh, Manar A. Nader, Marwa S. Zaghloul

**Affiliations:** 1 Department of Pharmacology and Toxicology, Faculty of Pharmacy, Mansoura University, Mansoura, Egypt; 2 Majmaah University, Majmaah, Saudi Arabia; 3 Department of Pharmacology and Toxicology, Faculty of Pharmacy, Mansoura National University, Gamasa, Egypt; 4 Future Studies and Risks Management & National Committee of Drugs, Academy of Scientific Research, Ministry of Higher Education, Elsayeda Zeinab, Egypt; 5 Faculty of Health Science Technology, Mansoura National University, Gamasa, Egypt

**Keywords:** acute pancreatitis, duloxetine, neuroinflammatory pain, PAR2, TRPV1

## Abstract

**Background:**

Acute pancreatitis (AP) is an inflammatory pancreatic disorder, often illustrated by neuropathic, inflammatory, and/or visceral pain. Pain is a medical indicator of pancreatic inflammation and growing evidence evokes that it exerts a significant role in AP progression via the widespread neuronal network surrounding the pancreas.

**Objectives:**

evaluate the possible defensive action of duloxetine (DUX), a serotonin/norepinephrine reuptake inhibitor (SNRI), in L-arginine (L-Arg)- provoked AP and multiple organ injury (MOI), and to explore the potential mechanism of action of DUX-mediated modulation of neuroinflammatory pain in AP.

**Methods:**

AP was induced in rats by intraperitoneal injection of L-Arg (100 mg/100 g) and two different doses of DUX (10 and 30 mg/kg) was examined.

**Results:**

DUX improved the histopathological architecture of the pancreas, liver, lung, and kidney. Additionally, it restored the normal levels of serum pancreatic enzymes, liver enzymes, and kidney biomarkers that were significantly elevated following L-Arg administration. In addition, DUX improved tissue antioxidant state. Moreover, DUX decreased the levels of transient receptor potential cation channel subfamily V member 1 (TRPV1), proteinase-activated receptor-2 (PAR2), substance P (SP), neurokinin, trypsin, leukotriene B4 (LTB4), signal transducer and activator of transcription 3 (STAT3) and significantly reduced TNF-α expression in the pancreatic tissues.

**Conclusion:**

DUX displayed analgesic, anti-inflammatory, and antioxidant effects. Therefore, DUX is a promising therapeutic candidate for the management of AP-related neuroinflammatory pain. The proposed analgesic and anti-inflammatory mechanisms of DUX may be linked to the modulation of SP related NK signaling and PAR2/TRPV1 pathways pointing to a possible role for these pathways in the observed beneficial action of DUX.

## 1 Introduction

Acute pancreatitis (AP) is an inflammatory disorder. It often presents neuropathic, inflammatory, and/or visceral pain (Beiriger et al., 2023). AP Acute pancreatitis (AP) can occur at any age, with a reported annual incidence ranging from 10 to 50 cases/100,000 individuals (Long, Jiang and Wu, 2022). The pathogenesis of AP is a dynamic process that starts with activation of the host immune response, leading to the release of proinflammatory cytokines and chemokine (Sundar et al., 2020). The leading causes of AP are gallstones and alcohol consumption, with gallstones responsible for approximately 60%–80% of cases. Additional causes include endoscopic retrograde cholangiopancreatography, drug-induced pancreatitis, metabolic disorders such as hypertriglyceridemia and hypercalcemia, autoimmune diseases, and idiopathic origins (Han et al., 2021).

Previous studies have revealed that mechanical factors, including pancreatic duct obstruction and elevated ductal and parenchymal pressures, are associated with pain in AP; however, these mechanisms alone are insufficient to fully explain the complexity of pain perception and signaling in AP (DiMagno, 1999; Karanjia et al., 1994). Subsequent studies have underscored the contributory role of neurogenic inflammation in the pathogenesis of AP-related pain (Liu, Shenoy and Pasricha, 2011). Consequently, pain research in animal models of AP has increasingly shifted from a predominant mechanical paradigm toward an emphasis on neurogenic inflammatory mechanisms (Steinhoff et al., 2014; Vera-Portocarrero and Westlund, 2005).

Noxious stimuli of pancreatic tissues lead to the production and release of several mediators, like trypsin, leukotriene B4 (LTB4) and protons. Such molecules are capable of engage diverse pain-related receptors and ion channels expressed on peripheral nerve terminals, including members of the transient receptor potential channels (TRP) family, e.g.,: TRP vanilloid 1 (TRPV1) and protease activated receptor 2 (PAR2). Neuropeptides including substance P (SP) and calcitonin-gene-related peptide (CGRP) were released triggered by peripheral nerve terminals within the pancreas, causing chemokine discharge from pancreatic acinar cells, plasma extravasation from venules, and interstitial edema within arterioles. Collectively, these alterations comprise neurogenic inflammation (Barreto et al., 2021).

The ensuing inflammatory response increases spinal cord excitability, thereby intensifying nociceptive signals arising from peripheral tissues. Similarly, a reciprocal interaction between damaged pancreatic tissue and activated neurons forms a self-perpetuating “auto-amplification loop” that connects inflammation and pain throughout the progression of AP (Liddle and Nathan, 2004; Vera-Portocarrero and Westlund, 2005). After this pathogenic cycle surpasses critical limits, it may trigger a sustained systemic inflammatory response syndrome, resulting in irreversible numerous organ injuries, and consequently a significant increase in mortality risk (Barreto et al., 2021).

Pain control has attracted increasing interest for the management of AP (Beiriger et al., 2023). It is the most challenging and characteristic symptom in acute and chronic pancreatitis. In a prospective study, approximately 77% of patients reported pain. Those experiencing pain had marked reductions in quality of life, and over 25% were on disability benefits (Szabolcs et al., 2006). Analgesics, opioids, and non-opioids are usually used to control pain in patients with AP but are associated with side effects and may even exacerbate AP (Wu et al., 2023). Consequently, the development of novel medical interventions and routines for pain management in patients with AP is necessary.

Improved management relies on the deeper understanding of the mechanisms underlying neuroinflammatory visceral pain, a topic that has recently progressed with the establishment of robust animal models and reliable experimental approaches for assessing sustained pancreatic pain. L-Arginine (L-Arg)-induced AP is an animal model of severe necrotizing AP. This model is extremely reproducible, noninvasive, and generates dose-dependent acinar necrosis, making it ideal for investigating the pathogenesis of AP (Szabolcs et al., 2006).

Duloxetine (DUX), a powerful serotonin/norepinephrine reuptake inhibitor (SNRI), is used in the management of major depressive disorders, painful diabetic neuropathy, and urinary incontinence (Park and Lee, 2018). Numerous preclinical studies have shown that DUX diminishes pain in a variety of insistent, neuropathic, and inflammatory pain models (Park and Lee, 2018). Hence, we speculated that DUX might have an ameliorative effect against AP-associated pain. The existing work was constructed to evaluate the possible defensive action of DUX in L-Arg-provoked AP and multiple organ injury (MOI), and to explore the potential mechanism of action of DUX-mediated modulation of neuroinflammatory pain in AP.

## 2 Materials and methods

### 2.1 Animals

Thirty-eight adult male Sprague–Dawley rats (190–240 g) from the MERC, Faculty of Medicine, Mansoura University, Egypt. The animals were kept in standard cages under regulated environmental conditions (22 °C ± 2 °C, 50%–60% relative humidity, and a 12-h light/dark cycle) with unrestricted access to standard chow and water. Following a 1-week acclimatization, rats were randomly distributed among the experimental groups. All procedures were approved from the Research Ethics Committee of the Faculty of Pharmacy, Mansoura University (approval number: MU-ACUC [PH.PHD.22.10.3]) and conducted in accordance with the National Institutes of Health Guide for the Care and Use of Laboratory Animals.

### 2.2 Drugs and chemicals

L-Arg was purchased from Sigma-Aldrich Chemical Co. (St. Louis, MO, USA). DUX was purchased from Lilly (Indianapolis, IN, USA). Both L-Arg and DUX were thawed in 0.9% saline and administered intraperitoneally (i.p.) to the rats. Reagents of the highest available purity were used throughout the study.

### 2.3 Investigational procedure

**FIGURE 1:**
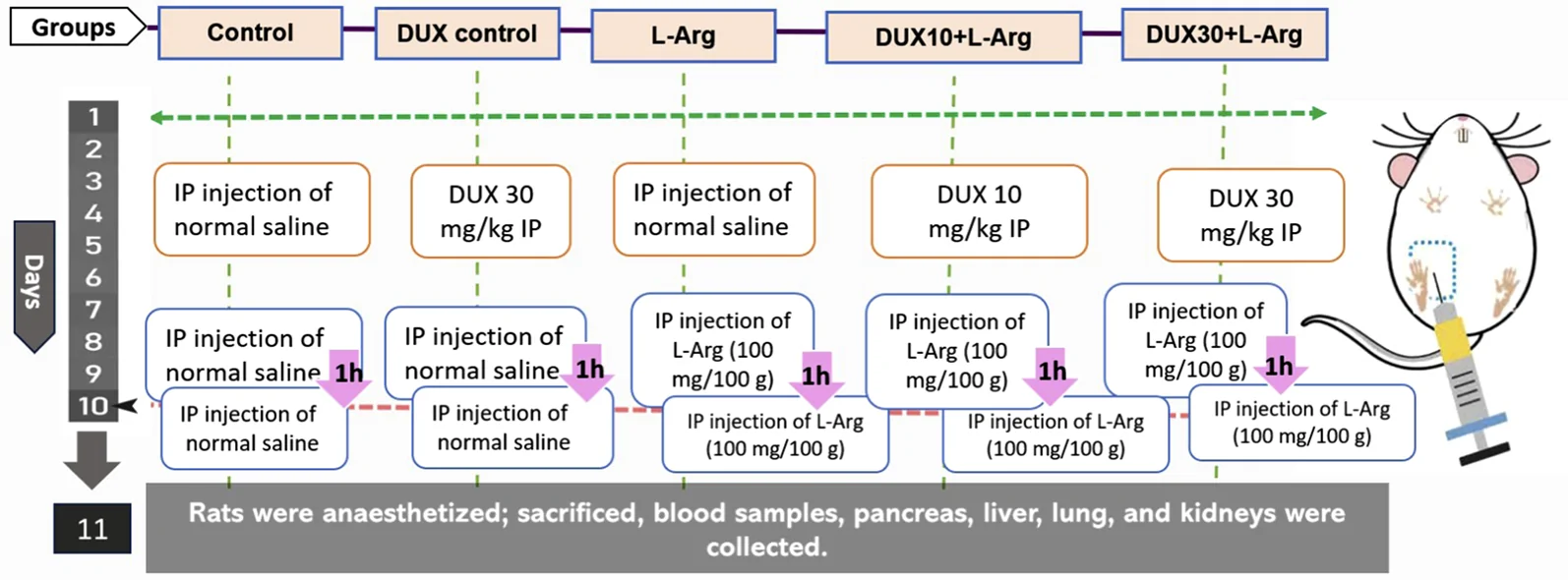
Experimental design and treatment protocol. Duloxetine (10 or 30 mg/kg, i. p.) or normal saline was administered once daily for 10 days. Acute pancreatitis was induced on day 10 by two intraperitoneal injections of L-arginine (100 mg/100 g each), given 1 h apart. Control animals received normal saline instead of L-arginine. On day 11, animals were anesthetized and euthanized, and blood and tissue samples (pancreas, liver, lungs, and kidneys) were collected for subsequent analyses.

Following period of acclimatization, age-matched rats with comparable body weights were randomly assigned into five groups and treated according to the protocols listed in Table 1 and Figure 1. The number of animals per group was selected based on previous studies employing similar L-Arg-induced AP models. Additional animals were initially included to compensate for the anticipated mortality associated with this severe experimental model. The selected duloxetine doses (10 and 30 mg/kg) were chosen based on previous experimental studies demonstrating their efficacy and safety in rodent models and to evaluate potential dose-dependent protective effects (Kim et al., 2017; Mannari et al., 2008; Mohammadi et al., 2019). 24 h after the final L-Arg boosting, rats were deeply anesthetized with thiopental sodium (40 mg/kg, i. p.). Blood samples (1.5 mL) were collected from the retro-orbital venous plexus for biochemical analyses. Animals were subsequently euthanized by cervical dislocation under deep anesthesia in accordance with the approved Animal Care and Use Committee (ACUC) protocol. Death was confirmed prior to tissue harvesting, after which pancreatic, left lateral liver lobe, left lung, and left kidney tissues were collected for histopathological and molecular analyses. Blood samples were withdrawn from the retro-orbital plexus, centrifuged at 5000 rpm for 15 min, and the resulted sera were subsequently preserved at −80 °C for subsequent serum biochemical analysis. Portion of the pancreas, left lateral liver lobe, left lung, and left kidney were fixed in 10% neutral buffered formalin for histopathological and immunohistochemical (IHC) examination. The remaining tissues (pancreas, median liver lobe, right lung, and right kidney) were homogenized in ice-cold phosphate buffer (0.01 M, pH 7.4) to obtain 10% w/v homogenates then centrifuged at 3000 rpm for 20 min at 4 °C, and the supernatants were collected and stored at −80 °C for subsequent assays.

**TABLE 1:** Experimental protocol.

Treatment protocol	Groups
Rats were injected with 0.9% normal saline (i.p.) for 10 days	Group I (Control) (n = 7)
Rats were administered DUX 30 mg/kg (i.p.) for 10 days	Group II (DUX Control) (n = 7)
Rats were injected with L-Arg dissolved in 0.9% saline (100 mg/100 g, i.p.) twice to create AP, with an interval of 1 h (Gulturk et al., 2021)	Group III (L-Arg) (n = 10)
Rats received DUX 10 mg/kg (i.p.) for 10 days prior to injection of 100 mg/100 g L-Arg twice with an interval of 1 h	Group IV (DUX10+L-Arg) (n = 7)
Rats received DUX 30 mg/kg (i.p.) for 10 days prior to injection of 100 mg/100 g L-Arg twice with an interval of 1 h	Group V (DUX30+L-Arg) (n = 7)

### 2.4 Spectrophotometric measurements

#### 2.4.1 Assessment of serum biochemical markers reflecting pancreatic, hepatic, renal, and pulmonary function

Serum amylase, lipase, alanine aminotransferase (ALT), aspartate aminotransferase (AST), creatinine (SCr), blood urea nitrogen (BUN), lactate dehydrogenase (LDH), and total protein (TP) levels were assessed spectrophotometrically using available kits. The absorbance was recorded using a spectrophotometer (Labomed, Los Angeles, USA) (Table 2).

**TABLE 2:** Spectrophotometric, ELISA, And IHC kits.

Kit manufacturers	Techniques	Kits
MDSS, Germany	Spectrophotometry	Amylase and lipase kits
Agappe Hills, India	Spectrophotometry	Alanine aminotransferase (ALT) kit Aspartate aminotransferase (AST) kit
SPINREACT, S.A./S.A.U Ctra.Santa Coloma, Spain	Spectrophotometry	Serum creatinine (SCr) kit Blood urea nitrogen (BUN) kit
Sedmed Diagnostics, India	Spectrophotometry	Lactate dehydrogenase (LDH) kit
Biodiagnostic, Egypt	Spectrophotometry	Total protein kit
Biodiagnostic, Egypt	Spectrophotometry	Malondialdehyde (MDA) kit
Biodiagnostic, Egypt	Spectrophotometry	Total antioxidant capacity (TAC) kit
Cat. No. OKEH03613, Aviva system biology, USA	ELISA	Transient receptor potential cation channel subfamily V member 1 (TRPV1) kit
Cat. No. NBP3-06968, biotechne®, USA	ELISA	Proteinase-activated receptor-2 (PAR2) kit
Cat. No. E-EL-0067, Elabscience®, USA	ELISA	Substance P (SP) kit
Cat. No. ABIN772124, antibodies-online.com, USA	ELISA	Neurokinin (NK) kit
Cat. No. ABIN6970901, antibodies-online.com, USA	ELISA	Trypsin kit
Cat. No. E-EL-0061, Elabscience®, USA	ELISA	Leukotriene B4 (LTB4) kit
Cat. No. ER0163, Fine Test®, China	ELISA	Signal transducer and activator of transcription 3 (STAT3) kit
Cat. No.GB11188, Service bio, 21 Olympia Avenue, Woburn, USA	IHC	Tumor necrosis factor-α (TNF-α) antibodies

#### 2.4.2 Evaluation of the oxidative and antioxidant status in tissues

Malondialdehyde (MDA), lipid peroxidation end-product, and total antioxidant capacity (TAC) were assessed using spectrophotometric available kits (Table 2).

### 2.5 Enzyme-linked immunosorbent assays (ELISA)

The pancreatic content of transient receptor potential cation channel subfamily V member 1 (TRPV1), proteinase-activated receptor-2 (PAR2), substance P (SP), neurokinin (NK), trypsin, leukotriene B4 (LTB4), and signal transducer and activator of transcription 3 (STAT3) were determined using commercially available ELISA kits (Table 2).

### 2.6 Histopathological examination

#### 2.6.1 Haematoxylin and Eosin (H&E) staining

Formalin-preserved sections of the pancreatic, hepatic, pulmonary, and renal tissues were placed in paraffin blocks. Thin sections (5 μm) were sliced and stained with H&E to evaluate any histopathological alterations. Histopathological abrasions were scored based on the criteria established in (Ceyhan et al., 2009; Janket et al., 2023; Tsuda et al., 2011).

#### 2.6.2 Immunohistochemical (IHC) analysis

Pancreatic TNF-α expression was evaluated via IHC analysis using the Avidin–Biotin Complex (ABC) technique as previously described (Wu et al., 2023). Fiji ImageJ software (version 1.51r; NIH, Maryland, USA) was utilized for quantification of % of positively stained areas. This approach generated three distinct digital channels corresponding to H&E, DAB, and a residual image. For quantitative assessment, five randomly selected microscopic fields (each measuring 200 × 200 µm) were analyzed per slide.

### 2.7 Statistical analysis

Statistical analysis was performed using GraphPad Prism version 8.0.1 (San Diego, CA, USA). Data normality was assessed with the Shapiro–Wilk test. Normally distributed data were analyzed using one-way ANOVA followed by Tukey–Kramer *post hoc* test for multiple comparisons and are expressed as mean ± SEM. Non-parametric scoring data are presented as median ± IQR and were analyzed using the Kruskal–Wallis test followed by Dunn’s *post hoc* test. A P value <0.05 was considered statistically significant.

## 3 Results

Current data revealed insignificant changes in any measured parameters concerning the normal control and DUX control groups. Mortality was observed exclusively in the L-arg group (40%), whereas no deaths occurred in the normal control, DUX control, or DUX-treated groups during the study period. Nevertheless, the study was not designed to evaluate survival as a primary outcome.

### 3.1 DUX deceased the elevated serum amylase, lipase, and LDH levels induced by L-Arg

**FIGURE 2:**
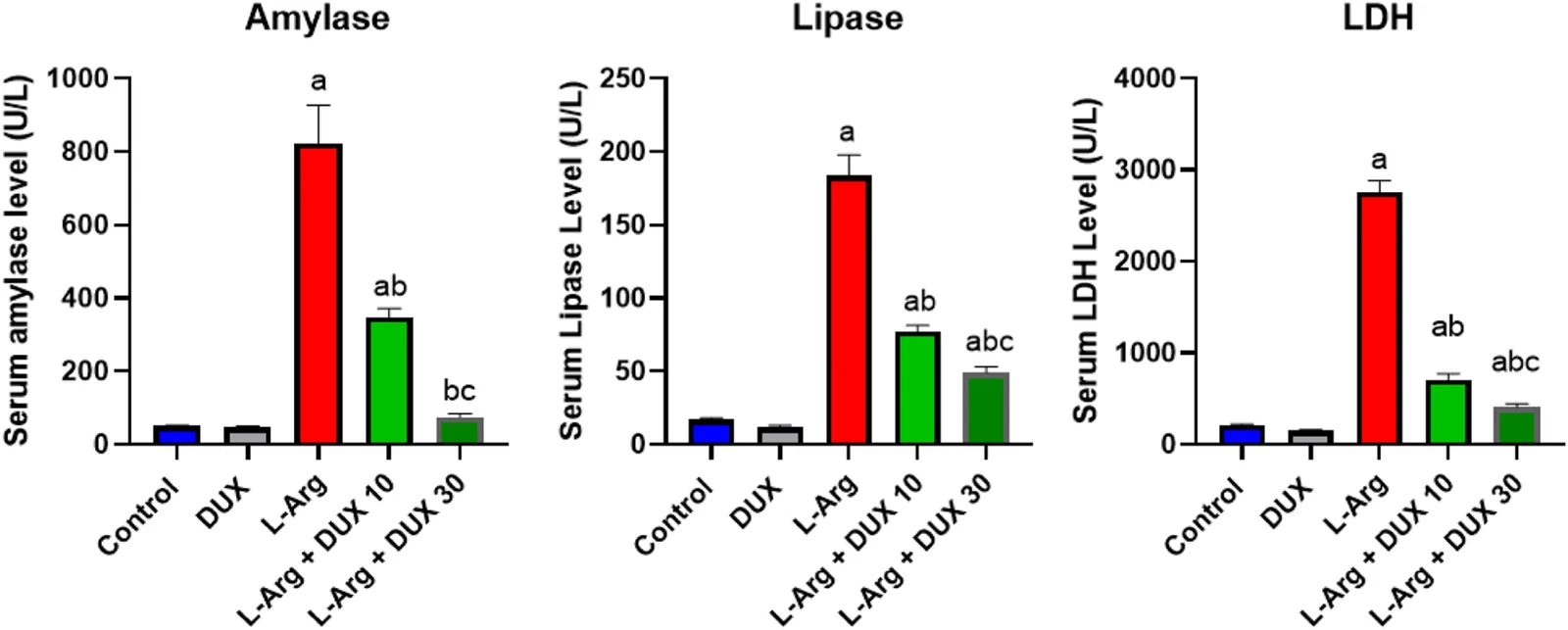
Effect of duloxetine (DUX) on serum amylase, lipase, and lactate dehydrogenase (LDH) levels in L-arginine (L-Arg) injected rats. Results are expressed as mean ± SEM (n = 6 for L-Arg group; n = 10 for others). ^a^ ^b^ ^c^ vs*.* the control, L-Arg, and L-Arg + DUX10 groups, respectively, as determined by one-way ANOVA with Tukey-Kramer multiple comparison *post hoc* tests (*P* < 0.05).

A noteworthy elevation in the levels of serum amylase, lipase, and LDH was detected in the L-Arg-treated group when matched the control group. DUX (10 mg/kg) treatment caused a substantial lowering in amylase, lipase, and LDH levels, comparative to the L-Arg group. On the other hand, their levels were remained meaningfully dissimilar to the control group. Conversely, DUX (30 mg/kg) administration in rats triggered a marked reduction in the amylase, lipase, and LDH levels in serum compared to L-Arg group, but there was still a significant difference in serum lipase and LDH levels between the DUX 30 and control groups. In addition, DUX 30 significantly lowered serum levels of amylase, lipase, and LDH compared with DUX 10 (Figure 2).

### 3.2 DUX decreased the elevated serum liver enzymes and total protein (TP) levels induced by L-Arg

**FIGURE 3:**
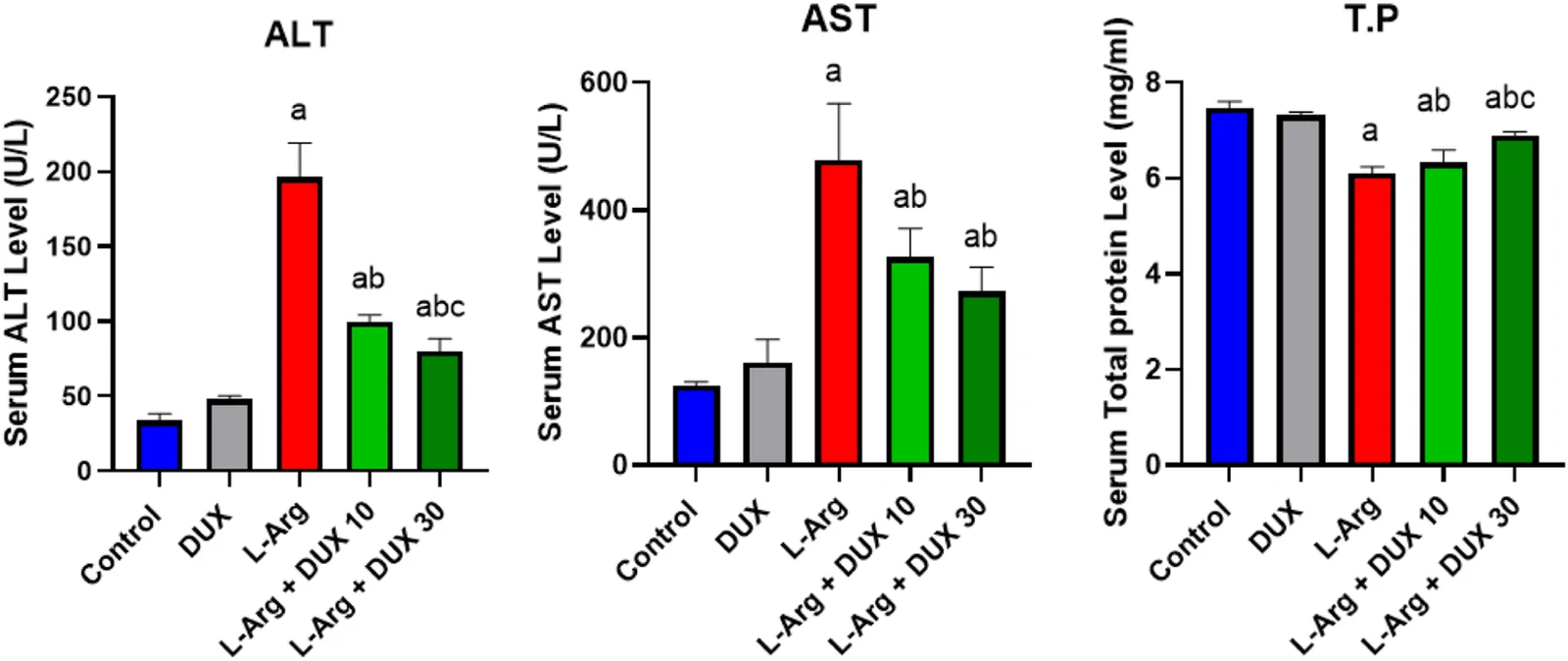
Effect of duloxetine (DUX) on serum alanine aminotransferase (ALT), aspartate aminotransferase (AST), and total protein (TP) levels in L-arginine (L-Arg) injected rats. Results are expressed as mean ± SEM (n = 6 for L-Arg group; n = 10 for others). ^a^ ^b^ ^c^ vs*.* the control, L-Arg, and L-Arg + DUX10 groups, respectively, as determined by one-way ANOVA with Tukey-Kramer multiple comparison *post hoc* tests (P < 0.05).

As shown in Figure 3, a noteworthy increase in the serum alanine aminotransferase (ALT), aspartate aminotransferase (AST) levels in serum, with a reduction in TP levels, was observed in L-Arg-administered rats compared to the control group. Nevertheless, rats treated with DUX 10 or DUX 30 showed dose dependent amelioration in serum ALT, AST, and TP levels relative to the L-Arg-injected group. However, a substantial dissimilarity among DUX 10, DUX 30 and the control group was noticed. In addition, pretreatment with DUX 30 ameliorated serum ALT and TP levels comparative to DUX 10-treated animals.

### 3.3 DUX mitigated the elevated kidney function biomarkers induced by L-Arg

**FIGURE 4:**
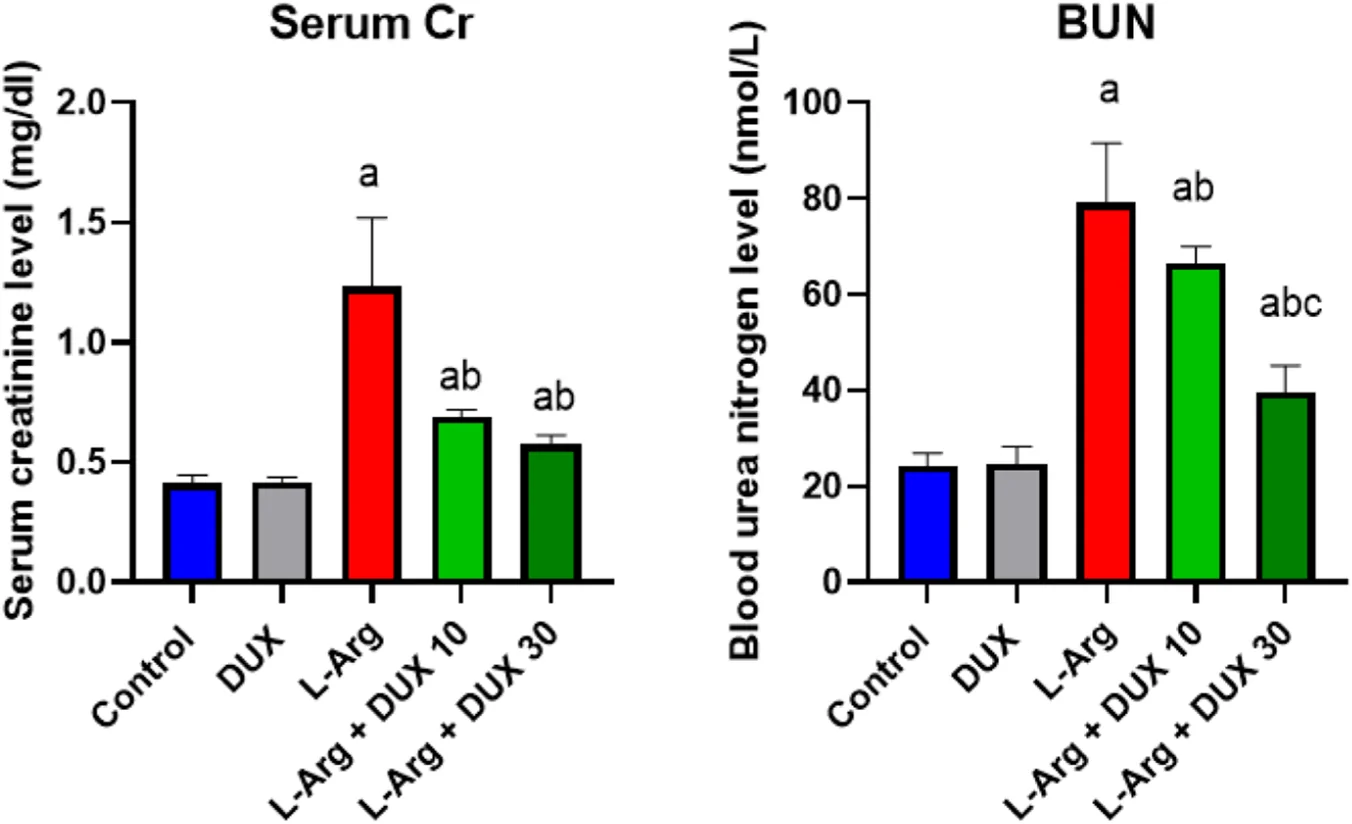
Effect of duloxetine (DUX) on serum creatinine (SCr) and blood urea nitrogen (BUN) in acute pancreatitis (AP) and multiple organ injury (MOI) induced by L-arginine in rats. Data are expressed as mean ± SEM, n = 6 for L-Arg group; n = 10 for other groups. L-Arg (100 mg/100 g, i.p.); DUX (10 or 30 mg/kg, i.p.); SEM: standard error of the mean. The values of ^a^, ^b^, ^c^ are significantly different from those of the control, L-Arg, and the (L-Arg + DUX 10) groups, respectively. This was determined using one-way ANOVA followed by Tukey-Kramer multiple comparisons post-hoc tests (P < 0.05).

As shown in Figure 4, L-Arg triggered a noteworthy rise in serum creatinine (SCr) and blood urea nitrogen (BUN) levels comparative to control rats. In contrast, DUX10 administration led to noteworthy lessening in SCr and BUN relative to L-Arg group, but still significantly higher than control rats. DUX 30 pretreatment significantly diminished SCr and BUN relative to L-Arg group, but still significantly higher than the control group. Moreover, DUX 30 significantly decreased the serum level of BUN compared to DX10.

### 3.4 DUX attenuated the L-Arg induced oxidative stress and improved the tissue anti-oxidant status

In L-Arg-injected animals, A noteworthy upsurge in malondialdehyde (MDA) levels in pancreatic, hepatic, pulmonary, and kidney tissues with a marked decrease in total antioxidant capacity (TAC) levels in the previously mentioned tissues compared to the control group was noticed. As matched to the L-Arg-treated group, DUX 10 group exhibited significant decrease in MDA content in the previous tissues in comparison with the L-Arg group and significantly elevated TAC| content in the previously mentioned tissues.

Both markers in all measured tissues were significantly higher than the levels in the control group. Conversely, oral ingestion of DUX 30 led to a noteworthy lessening in MDA content in the previously mentioned tissues and drastically augmented TAC content compared to the L-Arg group, but both markers in all measured tissues were still significantly higher than in the control group. Interestingly, DUX 30 significantly decreased MDA content in the pancreas, liver, lungs, and kidneys compared with DUX 10. In the same manner, DUX 30 significantly increased the level of TAC in the pancreas, liver, lungs, and kidneys compared to DUX 10 (Table 3).

**TABLE 3:** Effect of duloxetine (DUX) on oxidant/antioxidant level in different tissues in L-Arginine (L-Arg) injected rats.

Organ	Control	DUX	L-Arg	L-Arg +DUX 10	L-Arg +DUX 30
Malondialdehyde (MDA) (nmol/g tissue)					
Pancreas	330.80 ± 7.64	363.50 ± 9.82	1908.0 ± 52.42^a^	1138.0 ±35.35^a b^	650.7 ± 4.54^a b c^
Liver	250.00 ± 20.74	312.20 ± 4.36	1314.0 ± 68.92^a^	736.5 ±7.17^a b^	486.2 ± 5.95^a b c^
Kidney	218.30 ± 2.18	251.10 ± 4.36	793.3 ± 28.64^a^	508.7 ±2.52^a b^	342.8 ± 10.08^a b c^
Lung	153.90 ± 7.64	197.00 ± 3.27	633.2 ±7.87^a^	449.8 ±7.87^a b^	272.9 ± 8.26^a b c^
Total antioxidant capacity (TAC) (mM/L)					
Pancreas	0.63 ± 0.01	0.612 ± 0.004	0.12 ± 0.02^a^	0.33 ± 0.001^a b^	0.497 ± 0.020^a b c^
Liver	0.51 ± 0.01	0.517 ± 0.002	0.21 ± 0.01^a^	0.34 ± 0.005^ab^	0.467 ± 0.001^a b c^
Kidney	0.55 ± 0.01	0.519 ± 0.003	0.25 ± 0.01^a^	0.40 ± 0.004^ab^	0.479 ± 0.002^a b c^
Lung	0.58 ± 0.01	0.579 ± 0.013	0.27 ± 0.01^a^	0.38 ± 0.01^a b^	0.530 ± 0.010^a b c^

Data are presented as mean ± SEM (n = 6 for L-Arg group; n = 10 for all other groups).

^a^ ^b^ ^c^ vs*.* the control, L-Arg, and L-Arg + DUX10 groups, respectively, as determined by one-way ANOVA, followed by Tukey-Kramer multiple comparison *post hoc* tests (P < 0.05).

### 3.5 DUX ameliorated the histopathological alterations in the pancreas, liver, pulmonary, and renal tissues induced by L-Arg

#### 3.5.1 Pancreatic tissues

**FIGURE 5:**
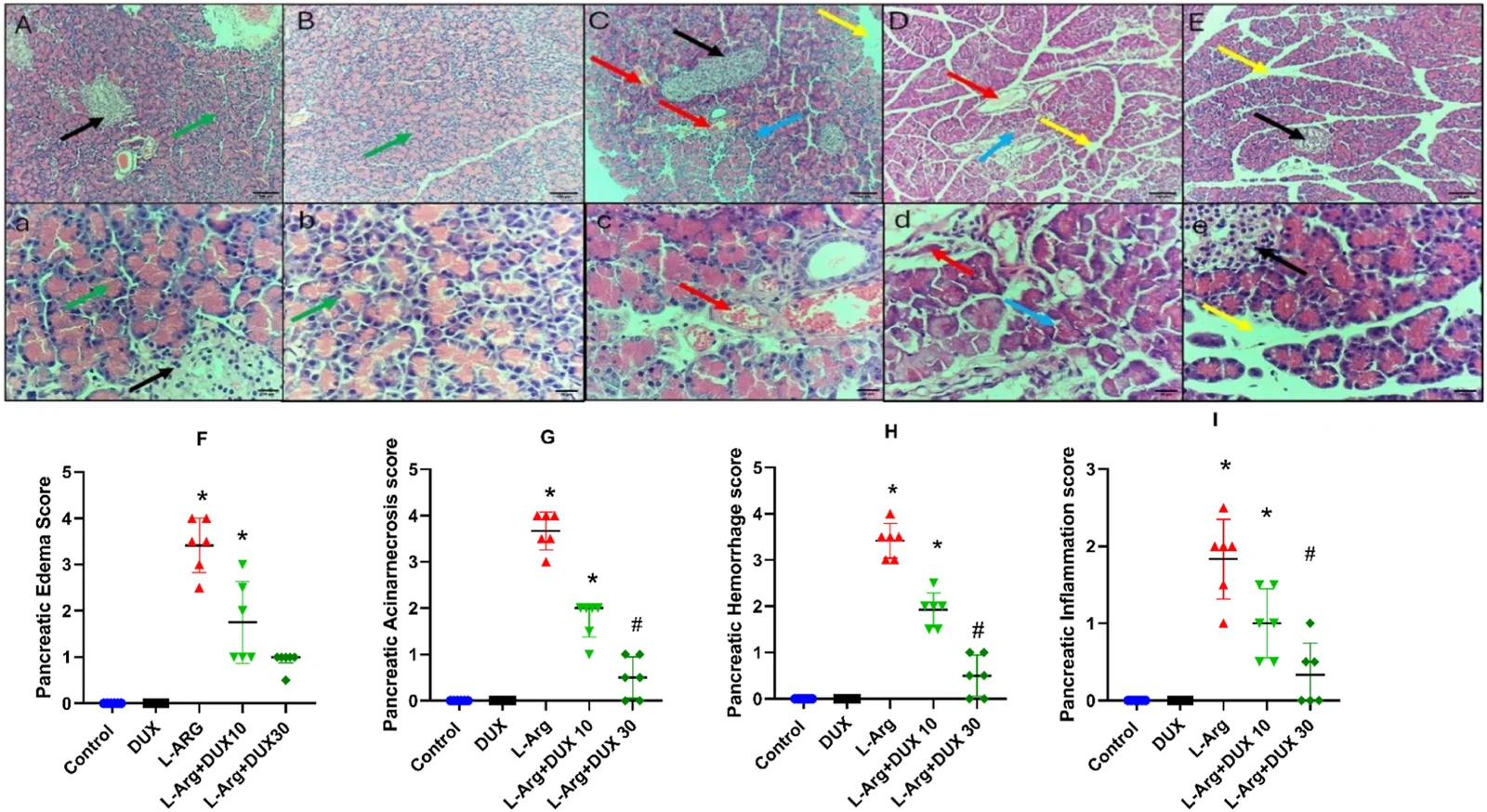
DUX ameliorated the histopathological alterations in the pancreas tissues induced by L-Arg. **(A,a)** Control group, **(B,b)** DUX control group, **(C,c)** L-Arg group, **(D,d)** L-Arg + DUX 10 group and **(E,e)** L-Arg + DUX 30 group. **(F)** Edema scores, **(G)** Acinar necrosis scores, **(H)** Hemorrhage scores and **(I)** Distribution of inflammation scores. Green arrow: presence of exocrine acini; Black arrow: islets of Langerhans; blue arrow: cellular injury; yellow arrow: disturbance of normal architecture; red arrow: vascular congestion. Upper panels were obtained at ×10 magnification (100 µm scale bar), while those of the lower panels were obtained at ×40 magnification (20 µm scale bar). Data are presented as median ± IQR. * # vs*.* the control and L-Arg-treated group, as found with Kruskal–Wallis test with Dunn multiple comparison test (P < 0.05).

The control group (Figures 5A,a) and DUX group (Figures 5B,b) maintained normal pancreatic architecture, with intact exocrine acini and islets of Langerhans. In contrast, the L-Arg group (Figures 5C,c), exhibited disruption of exocrine acinar structure, vascular congestion, cellular damage, and infiltration of the inflammatory cells. The DUX10 group (Figures 5D,d) displayed moderate architectural disruption, congested blood vessels, and cellular injury. Notably, the DUX30 + L-Arg group (Figure 4E,e) demonstrated only minimal disturbance of acinar architecture, with no detectable cellular damage or vascular congestion. Semi-quantitative analysis of pancreatic edema, acinar necrosis, hemorrhage, and inflammation (Figures 5F–I respectively) revealed significant increases in the L-Arg group compared to controls. Treatment with DUX10 and DUX30 substantially reduced these pathological scores relative to the L-Arg group, although differences remained when compared with the normal control group.

#### 3.5.2 Hepatic tissues

**FIGURE 6:**
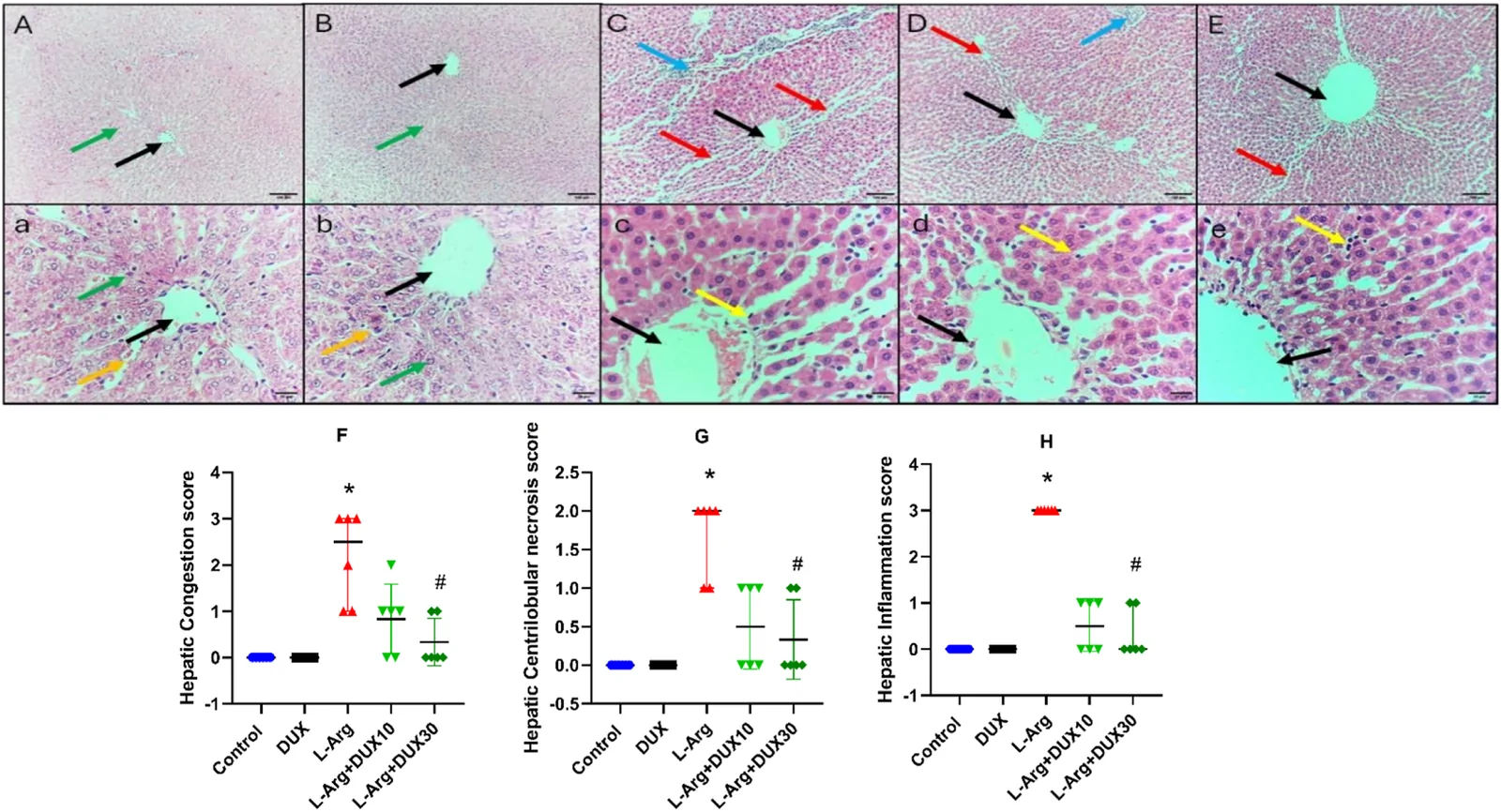
DUX ameliorated the histopathological alterations in the hepatic tissues induced by L-Arg. **(A,a)** Control group, **(B,b)** DUX control group, **(C,c)** L-Arg group, **(D,d)** L-Arg + DUX 10 group and **(E,e)** L-Arg + DUX 30 group. **(F)** Congestion scores, **(G)** Centrilobular scores and **(H)** Inflammation scores. Green arrow: hepatocytes; black arrow: central vein; orange arrow: sinusoids; yellow arrow: Kupffer cells; blue arrow: inflammatory cell infiltration; red arrow: congested sinusoids. Upper panels were obtained at ×10 magnification (100 µm scale bar), while those of the lower panels were obtained at ×40 magnification (20 µm scale bar). Data are presented as median ± IQR. * # vs*.* the control and L-Arg-treated group, as found with Kruskal–Wallis test with Dunn multiple comparison test (P < 0.05).

Liver sections from the normal control group (Figures 6A,a) and the DUX control group (Figures 6B,b) demonstrated a normal hepatic architecture characterized by polyhedral hepatocytes arranged in cords separated by blood sinusoids containing Kupffer cells surrounding the central vein. In the L-Arg-treated group (Figures 6C,c), marked sinusoidal congestion, infiltration of the inflammatory cells, and increased the Kupffer cell activity were observed. The DUX 10 group (Figures 6D,d) exhibited moderate sinusoidal congestion with elevated Kupffer cell activity and minimal inflammatory infiltration. In contrast, the DUX 30 group (Figures 6E,e) showed only mild sinusoidal congestion without inflammatory cell infiltration. Moreover, L-Arg administration significantly increased portal inflammation and hepatic centrilobular necrosis compared with the control group. Treatment with DUX at 10 and 30 mg noticeably reduced portal inflammation scores relative to the L-Arg group (Figure 6F–H).

#### 3.5.3 Lung tissues

**FIGURE 7:**
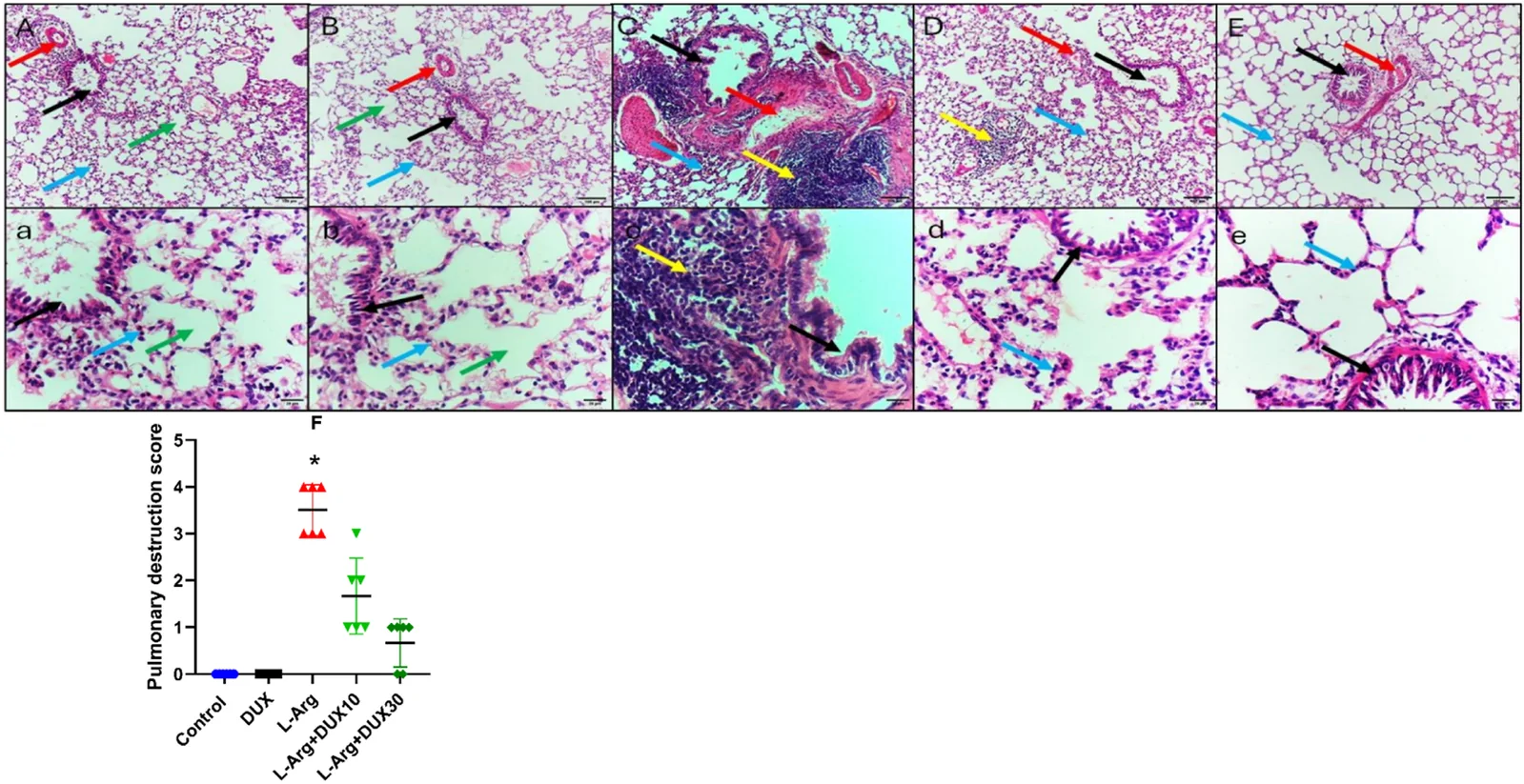
DUX ameliorated the histopathological alterations in the pulmonary tissues induced by L-Arg. **(A,a)** Control group, **(B,b)** DUX control group, **(C,c)** L-Arg group, **(D,d)** L-Arg + DUX 10 group and **(E,e)** L-Arg + DUX 30 group. **(F)** Pulmonary destruction scores. Green arrow: alveoli; black arrow: bronchioles; blue arrow: interalveolar septum; Yellow arrow: inflammatory cell infiltration; red arrows: bronchial arterioles. Upper panels were obtained at ×10 magnification (100 µm scale bar), while those of the lower panels were obtained at ×40 magnification (20 µm scale bar). Data are presented as median ± IQR. * # vs*.* the control and L-Arg-treated group, as found with Kruskal–Wallis test with Dunn multiple comparison test (P < 0.05).

Lung sections from the normal control group (Figures 7A,a) and the DUX control group (Figures 7B,b) displayed normal pulmonary architecture, with well-defined alveoli separated by thin interalveolar septa along with normal bronchioles and bronchial arterioles. The L-Arg-injected group (Figures 7C,c) showed marked distortion of lung architecture, characterized by thickened interalveolar septa, reduced alveolar spaces, inflammatory cell infiltration within the septa and around bronchi, and congested, thickened pulmonary arterioles. In the DUX 10 group (Figures 7D,d), moderate thickening of the interalveolar septa with smaller alveoli, inflammatory cell infiltration, and congested pulmonary arterioles were observed. Meanwhile, the DUX 30 group (Figures 7E,e) demonstrated nearly normal pulmonary septa with only minimal thickening of the pulmonary arteriolar wall. Histopathological scoring revealed a significant increase in lung tissue damage in the L-Arg group compared with the normal control. Treatment with DUX (10 and 30 mg) reduced the damage score relative to the L-Arg group; however, both groups still showed significant differences compared with the normal group (Figure 7F).

#### 3.5.4 Kidney tissues

**FIGURE 8:**
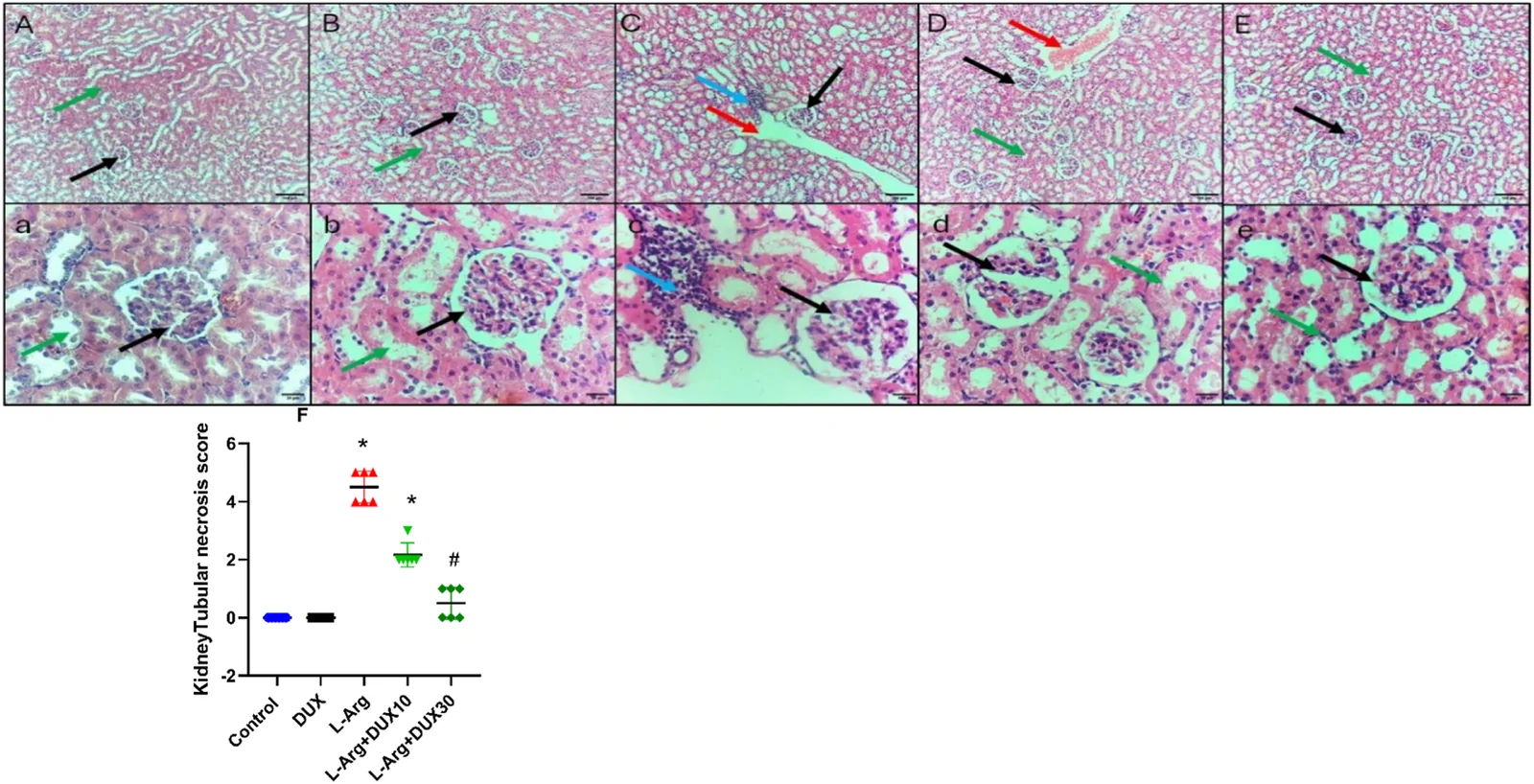
DUX ameliorated the histopathological alterations in the renal tissues induced by L-Arg. **(A,a)** Control group, **(B,b)** DUX control group, **(C,c)** L-Arg group, **(D,d)** L-Arg + DUX 10 group and **(E,e)** L-Arg + DUX 30 group. **(F)** Tissue tubular necrosis scores. Green arrow: renal tubules; black arrow: glomeruli; blue arrow: inflammatory cell infiltration; yellow arrow: swelling of tubular cells; red arrow: damaged tubular cells. Upper panels were obtained at ×10 magnification (100 µm scale bar), while those of the lower panels were obtained at ×40 magnification (20 µm scale bar). Data are presented as median ± IQR. * # vs*.* the control and L-Arg-treated group, as found with Kruskal–Wallis test with Dunn multiple comparison test (P < 0.05).

H&E-stained kidney sections from the control (Figures 8A,a) and DUX control groups (Figures 8B,b) displayed normal renal architecture, with appropriately sized glomeruli within Bowman’s space and intact proximal and distal tubules. Figure 8C, C revealed that injection of L-Arg disrupted the renal architecture, a glomerular shrinkage, expanded Bowman’s space, tubular epithelial cells degeneration and inflammatory cell infiltration were noticed.

In the DUX10 group (Figures 8D,d), moderate architectural distortion was observed, including glomerular shrinkage, expanded Bowman’s space, accumulation of the inflammatory cells, and tubular epithelial degeneration. The DUX30 group (Figures 8E,e) revealed only minimal collapsed glomeruli with a widened Bowman’s space and mild tubular epithelial changes. Tubular necrosis scoring indicated a significant increase in the L-Arg group compared to controls, whereas treatment with DUX10 and DUX30 significantly reduced necrosis scores relative to the L-Arg-treated group (Figure 8F).

### 3.6 Effect of DUX on pancreatic protein levels of trypsin, proteinase-activated receptor-2 (PAR2), transient receptor potential cation channel subfamily V member 1 (TRPV1), substance P, neurokinin, leukotriene B4 (LTB4), and signal transducer and activator of transcription 3 (STAT3)

**FIGURE 9:**
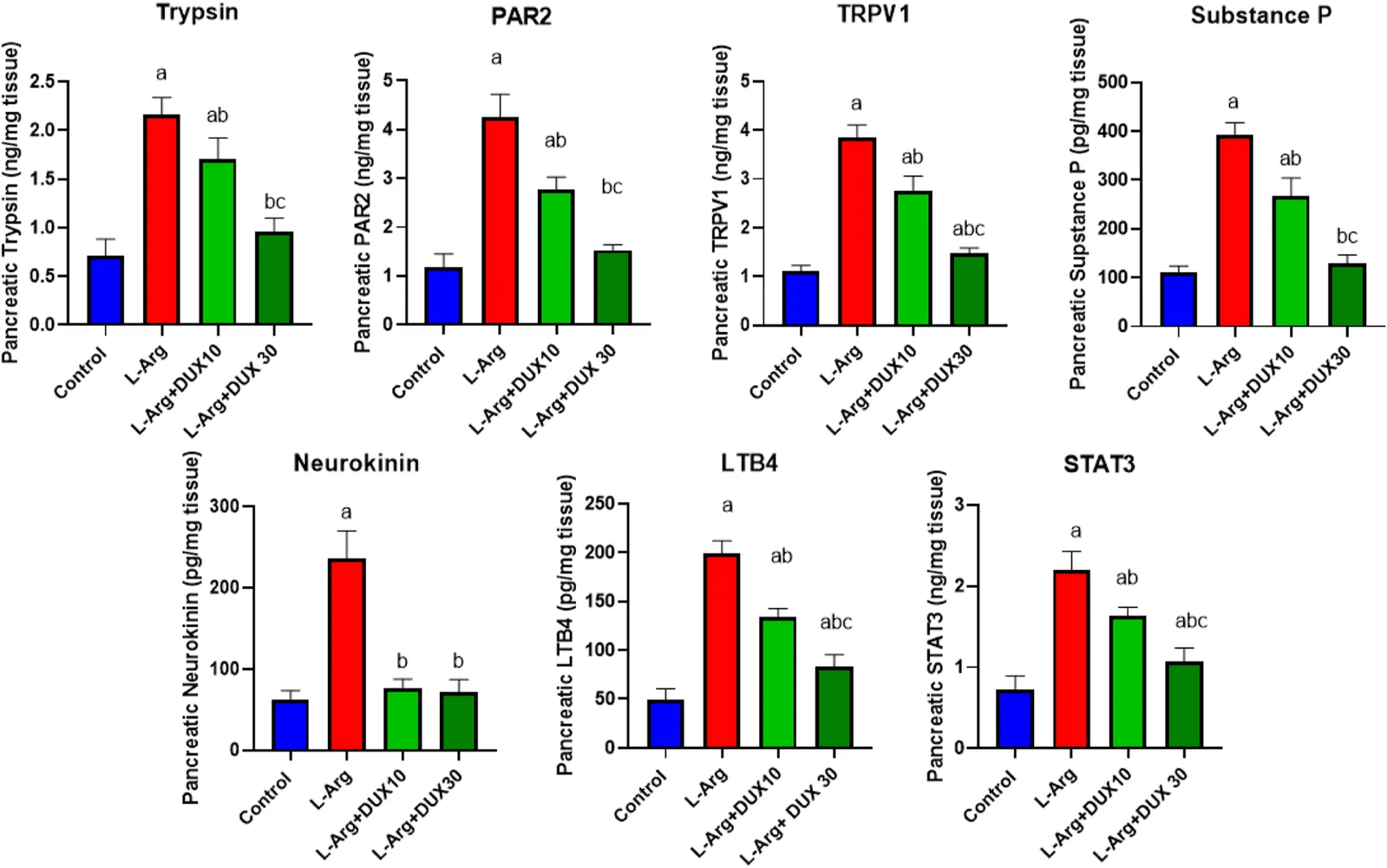
Effect of duloxetine (DUX) on trypsin, proteinase-activated receptor-2 (PAR2), transient receptor potential cation channel subfamily V member 1 (TRPV1), substance P, neurokinin, leukotriene B4 (LTB4), and signal transducer and activator of transcription 3 (STAT3) levels in L-arginine (L-Arg) injected rats. Data are presented as mean ± SEM (n = 6 for L-Arg group; n = 10 for others). ^a^ ^b^ ^c^ vs*.* the control, L-Arg, and L-Arg + DUX10 groups, respectively, as determined by one-way ANOVA with Tukey-Kramer multiple comparison *post hoc* tests (P < 0.05).

Figure 9 demonstrated that L-Arg inoculation triggered a considerable elevation in the pancreatic trypsin, PAR2, TRPV1, SP, neurokinin, LTB4, and STAT3 levels compared to control animals. Management with DUX 10 led to a marked decrease in previous markers compared to the L-Arg group. DUX 30 handling considerably lessened pancreatic levels of measured parameters when matched to L-Arg rats. Furthermore, it was noted that DUX 30 significantly decreased levels of trypsin, PAR2, TRPV1, SP, LTB4, and STAT3 compared with DUX 10.

### 3.7 DUX suppressed the elevated tumor necrosis factor-α (TNF-α) immune-expression in pancreatic tissues induced by L-Arg

**FIGURE 10:**
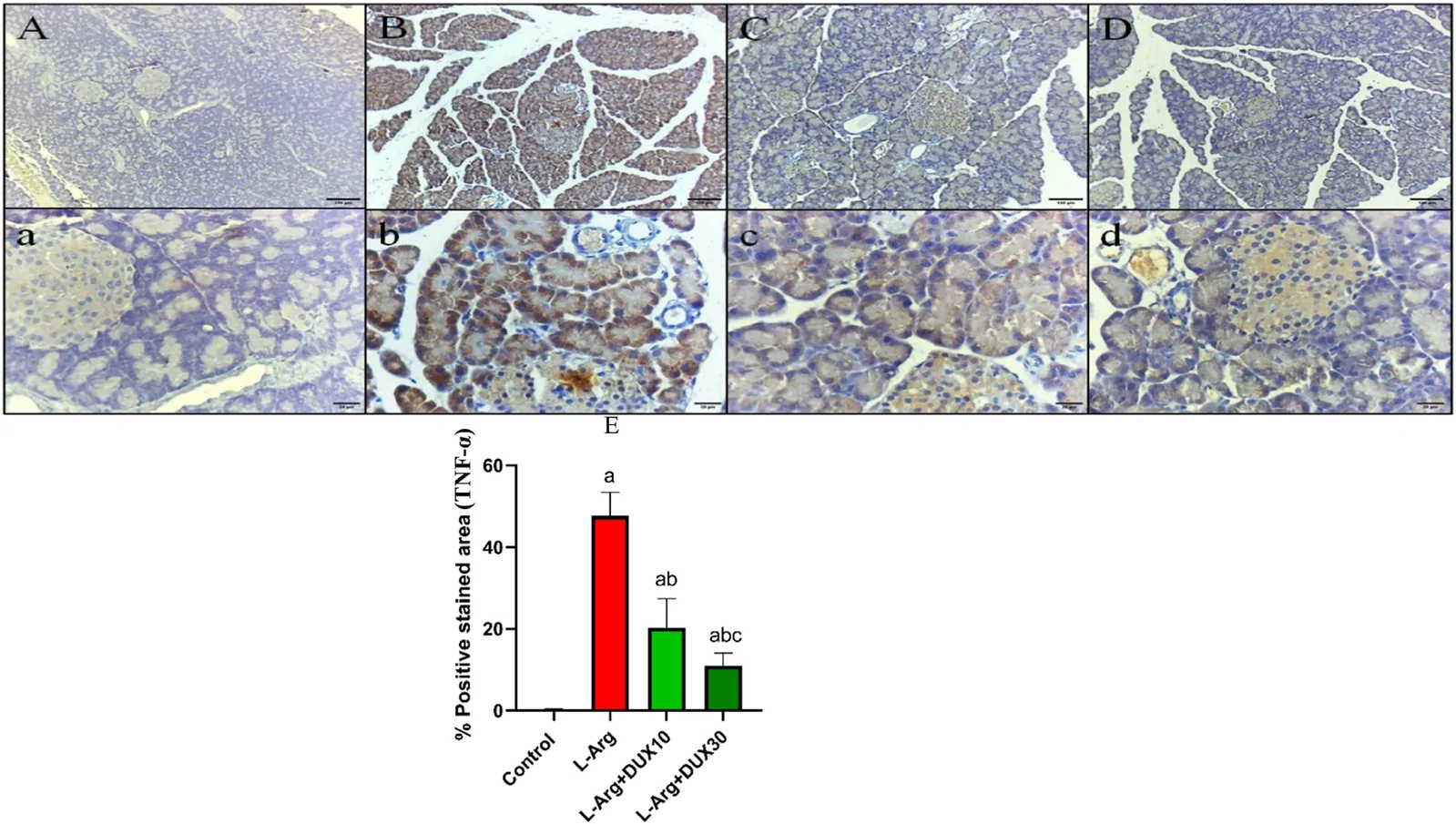
DUX suppressed the elevated tumor necrosis factor-α (TNF-α) immune-expression in pancreatic tissues induced by L-Arg. **(A,a)** Control group, **(B, B)** L-Arg treated group, **(C,C)** L-Arg + DUX 10 group, **(D,d)** L-Arge + DUX 30 group; **(E)** The percentage of positively immune-stained regions. Upper panel was captured at magnification ×10 (100 µm scale bar), while the lower panel was captured at x40 (20 µm scale bar). ^a^ ^b^ ^c^ vs*.* the control, L-Arg, and L-Arg + DUX10 groups, respectively, as determined by one-way ANOVA with Tukey-Kramer multiple comparison *post hoc* tests (P < 0.05).

Immunohistochemical staining for TNF-α in rat pancreatic tissue sections revealed no detectable immunoreactivity in the control group (Figures 9A,a). In contrast, the L-Arg group (Figures 10B,b) exhibited strong cytoplasmic expression in the exocrine acinar cells and moderate staining in the islets of Langerhans. The DUX 10 group (Figures 10C,c) demonstrated moderate cytoplasmic TNF-α expression in both exocrine acinar cells and the islets of Langerhans. Meanwhile, the DUX 30 group (Figures 9D,d) showed only mild cytoplasmic immunoreactivity in these pancreatic structures.

## 4 Discussion

Acute pancreatitis (AP) is characterized by complex neuroimmune crosstalk that amplifies inflammatory cascades and nociceptive transmission. Pain in AP comprises both nociceptive and neuropathic components driven by neurogenic inflammation, ion channel activation, and cytokine-mediated central sensitization (Beiriger et al., 2023; Liddle and Nathan, 2004; Wu and Han, 2023). The present study demonstrates that DUX confers marked protection against L-arginine–induced AP, attenuating pancreatic injury, systemic organ injury, and pain-related behavioral alterations through coordinated modulation of inflammatory, neurogenic, and oxidative pathways.

Biochemically and histologically, the L-Arg model reliably reproduced severe AP, evidenced by marked elevations in serum amylase and lipase, destruction of acinar architecture, inflammatory infiltration, vascular congestion, and multi-organ injury reflected by increased ALT, AST, creatinine, BUN, and LDH levels (Ateyya, Wagih and El-Sherbeeny, 2016; Nader and Wagih, 2017; Schmidt et al., 1992). These findings confirm the robustness and translational relevance of this model for evaluating therapeutic interventions targeting both pancreatic and systemic sequelae.

Proteinase-activated receptor-2 (PAR2), transient receptor potential cation channel subfamily V member 1 (TRPV1) levels were further determined by ELISA represent tissue protein expression and should not be interpreted as direct measures of receptor activation. Mechanistically, pancreatic pain is largely driven by neurogenic inflammation originating from T5–L2 dorsal root ganglia afferents innervating the pancreas (Candal, Reddy and Samra, 2025; Drewes et al., 2020). Activation of TRPV1 channels enhances Ca^2+^ influx and stimulates SP release, which binds NK1R and amplifies inflammatory signaling within pancreatic tissue (Jensen et al., 2017; Liddle and Nathan, 2004; Nakanishi, 1991; Vera-Portocarrero and Westlund, 2005). Upregulation of SP related NK1R signaling has been documented in experimental and clinical AP (Caberlotto et al., 2003; Han et al., 2021). In the present study, L-Arg significantly increased SP, NK, and TRPV1 expression, confirming activation of this neuroinflammatory axis. DUX markedly suppressed these mediators, indicating concomitant downregulation of the SP-related NK1R and TRPV1 pathways indicating disruption of the SP related NK1R–TRPV1 feed-forward loop that perpetuates pancreatic inflammation and pain. Protease-driven signaling represents an additional amplification pathway. Trypsin released during acinar injury activates PAR2 on nociceptive neurons, which is known to transactivates TRPV1 and enhances neuronal excitability (Nishimura et al., 2010; Olesen et al., 2017; Toma et al., 2000). Elevated pancreatic trypsin and PAR2 levels in AP animals confirm engagement of this cascade. Importantly, DUX significantly reduced both trypsin and PAR2 expression, suggesting modulation of protease-dependent nociceptive sensitization.

Previous studies have demonstrated that experimental acute pancreatitis may be associated with behavioral manifestations of pain, including mechanical hypersensitivity and thermal hyperalgesia. In the present study, however, behavioral nociceptive assessments were not conducted. Therefore, our findings are limited to biochemical and molecular alterations in pain-related pathways, including SP-, PAR2-, and TRPV1-associated signaling, and should not be interpreted as direct evidence of analgesic activity (Schwartz et al., 2011; Wick et al., 2006). Furthermore, while these mediators are biochemically linked, our current expression data are correlative; hence, the precise sequential hierarchy of the trypsin/PAR2/TRPV1/SP axis remains a proposed mechanism rather than a definitively proven pathway in this specific setting. Future studies incorporating pathway-specific inhibitors or gene-knockdown models, alongside behavioral endpoints, are needed to establish the causal relationships and functional significance of these molecular changes.

Leukotriene B4 (LTB4) further sustains TRPV1 activation by sensitizing pancreatic sensory neurons (Shahid et al., 2015; Vigna et al., 2011). Increased LTB4 levels observed in the AP group provide additional evidence of lipid mediator–driven neurogenic inflammation. The reduction of LTB4 by DUX indicates broader suppression of inflammatory lipid signaling contributing to nociceptive amplification.

Beyond peripheral mechanisms, transcriptional regulation via STAT3 plays a critical role in sustaining neuroinflammation and chronic pain states (Li et al., 2017; Tsuda et al., 2011). Increased STAT3 expression following L-Arg administration suggests activation of downstream inflammatory gene programs that reinforce central sensitization. DUX significantly attenuated STAT3 expression, highlighting its capacity to interfere with the overall expression of transcriptional machinery that links immune activation to persistent pain signaling.

Tumor necrosis factor-α (TNF-α), a master pro-inflammatory cytokine elevated in severe AP, contributes to neutrophil recruitment, endothelial activation, and tissue injury (Atarbashe and Abu-raghif 2020). Importantly, TNF-α also facilitates neuropathic pain by disrupting the perineural barrier and promoting axonal sensitization (Ceyhan et al., 2009). The marked increase in TNF-α observed in AP animals underscores its dual inflammatory and nociceptive role. DUX significantly reduced TNF-α levels, thereby attenuating both inflammatory progression and pain amplification.

Oxidative stress constitutes a pivotal link between tissue injury and nociceptive sensitization. Excess reactive oxygen species (ROS) enhance TRPV1 activity, disrupt mitochondrial function, and propagate inflammatory cascades (Demirdaş, Nazıroğlu and Övey İ 2017). Consistent with previous evidence demonstrating antioxidant effects of DUX via modulation of Ca^2+^ influx and ROS production (Demirdaş et al., 2017), our results show reduced MDA levels and restoration of total antioxidant capacity across pancreatic and remote tissues. This antioxidant action likely synergizes with anti-inflammatory and neurogenic modulation to confer organ protection and analgesia.

Pharmacologically, DUX acts as a serotonin and norepinephrine reuptake inhibitor, enhancing descending inhibitory pathways within the spinal cord (Hoshino, Obata and Saito, 2015; Ito et al., 2018). Increased synaptic 5-HT and NE strengthen endogenous analgesic circuits and counterbalance ascending nociceptive transmission. Thus, DUX integrates central neuromodulation with peripheral anti-inflammatory and antioxidative effects, resulting in comprehensive attenuation of AP-associated pain.

## 5 Conclusion

**FIGURE 11:**
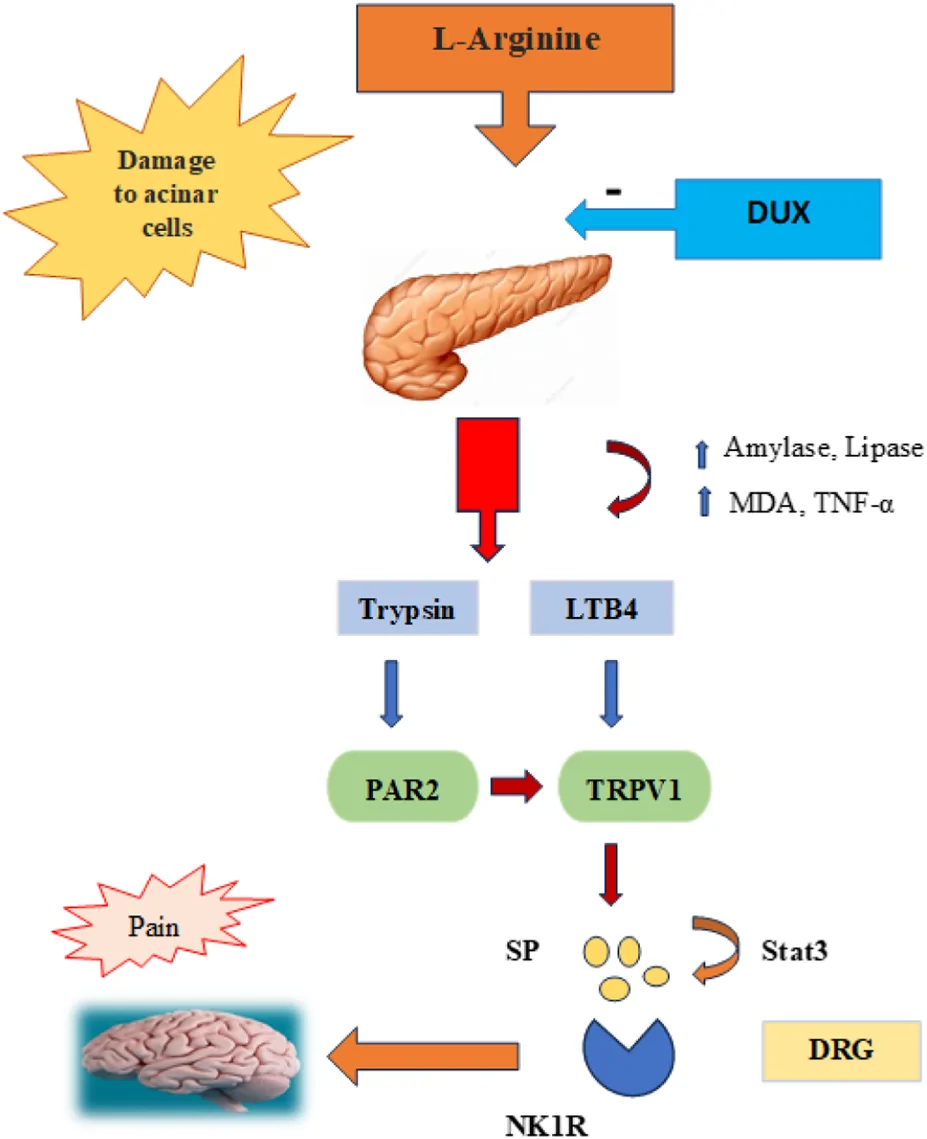
Schematic diagram outlining the effect of duloxetine (DUX) against L-arginine (L-Arg)-induced acute pancreatitis (AP) and multiple organ injury (MOI). LTB4: leukotriene B4; TRPV1: transient receptor potential cation channel subfamily V member 1; PAR2: proteinase-activated receptor-2; Stat 3: Signal transducer and activator of transcription 3; SP: Substance P; NK1R: Neurokinin 1 receptor; MDA: malondialdehyde; TNF-α: tumor necrosis factor- α; DUX: Duloxetine. DRG: dorsal root ganglion.

Collectively, our findings demonstrate that protective effects of DUX in experimental AP are associated with modulation of markers linked to SP/NK1R signaling, PAR2/TRPV1 activation, LTB4 sensitization, STAT3-activity, TNF-α liberation, and oxidative stress. As far as we know, this is the first study to demonstrate concurrent alteration in these interconnected pathways following DUX in experimental AP, supporting its possible repurposing as a multimodal therapeutic strategy targeting management of both pancreatic inflammation and pain; however, further mechanistic studies are required to establish causal pathway involvement (Figure 11).

## 6 Limitation

Despite the promising findings of the present study, several limitations should be acknowledged. First, the study was conducted using an acute L-Arg-induced AP model, which may not fully recapitulate the complexity of human AP. Second, the mechanistic conclusions are based primarily on alterations in pathway-related biomarkers and protein expression levels. Although changes in SP, PAR2, TRPV1, STAT3, TNF-α, and oxidative stress markers were observed, receptor activation and downstream signaling were not directly assessed, and no receptor antagonists or pathway-specific inhibitors were employed to establish causality. Furthermore, NK1R expression was not directly quantified, and PAR2/TRPV1 measurements reflected tissue protein expression rather than functional receptor activity. In addition, complementary techniques such as Western blotting, immunofluorescence, or gene expression analyses were not performed. In addition to, although additional animals were included to compensate for the anticipated mortality associated with the L-Arg model, a formal *a priori* power analysis was not conducted. Therefore, future studies incorporating larger sample sizes, mechanistic interventions, and comprehensive molecular analyses are warranted to further validate and extend the present findings. Also, an additional limitation is the absence of behavioral pain assessments. Although several nociception-related biomarkers, including SP, PAR2, and TRPV1, were evaluated, validated behavioral measures of pain sensitivity, such as von Frey, thermal nociception, or abdominal withdrawal tests, were not performed. Therefore, the present findings provide indirect evidence regarding pain-related mechanisms and do not establish a direct analgesic effect of duloxetine. Finally, an important limitation is that DUX was administered before the induction of acute pancreatitis. Consequently, the present experimental design evaluated the preventive (prophylactic) rather than the therapeutic effects of DUX. Therefore, the observed protective effects cannot be directly extrapolated to the treatment of established AP in clinical settings. Future studies employing post-induction treatment protocols are necessary to determine the therapeutic potential of duloxetine after disease onset.

## Data Availability

The raw data supporting the conclusions of this article will be made available by the authors, without undue reservation.
